# Molecular basis for sortase-catalyzed pilus tip assembly

**DOI:** 10.1128/mbio.01484-24

**Published:** 2024-08-02

**Authors:** Aadil H. Bhat, Chungyu Chang, Asis Das, Hung Ton-That

**Affiliations:** 1 Division of Oral & Systemic Health Sciences, School of Dentistry, University of California, Los Angeles, California, USA; 2 Department of Medicine, Neag Comprehensive Cancer Center, School of Medicine, University of Connecticut Health Center, Farmington, Connecticut, USA; 3 Department of Microbiology, Immunology & Molecular Genetics, University of California, Los Angeles, California, USA; 4 Molecular Biology Institute, University of California, Los Angeles, California, USA; The University of Kansas Medical Center, Kansas City, Kansas, USA

**Keywords:** *Actinomyces oris*, sortase, pilus assembly, tip pilin, secretion, cell wall anchoring, coaggregation

## Abstract

During pilus assembly within the Gram-positive bacterial envelope, membrane-bound sortase enzymes sequentially crosslink specific pilus protein monomers through their cell wall sorting signals (CWSS), starting with a designated tip pilin, followed by the shaft made of another pilin, ultimately anchoring the fiber base pilin to the cell wall. To date, the molecular determinants that govern pilus tip assembly and the underlying mechanism remain unknown. Here, we addressed this in the model organism *Actinomyces oris*. This oral microbe assembles a pathogenically important pilus (known as type 2 fimbria) whose shafts, made of FimA pilins, display one of two alternate tip pilins—FimB or the coaggregation factor CafA—that share a markedly similar CWSS. We demonstrate that swapping the CWSS of CafA with that of FimB produces a functional hybrid, which localizes at the pilus tip and mediates polymicrobial coaggregation, whereas alanine-substitution of the conserved FLIAG motif within the CWSS hampers these processes. Remarkably, swapping the CWSS of the normal cell wall-anchored glycoprotein GspA with that of CafA promotes the assembly of hybrid GspA at the FimA pilus tip. Finally, exchanging the CWSS of the *Corynebacterium diphtheriae* shaft pilin SpaA with that of CafA leads to the FLIAG motif-dependent localization of the heterologous pilus protein SpaA at the FimA pilus tip in *A. oris*. Evidently, the CWSS and the FLIAG motif of CafA are both necessary and sufficient for its destination to the cognate pilus tip specifically assembled by a designated sortase in the organism.

**IMPORTANCE:**

Gram-positive pili, whose precursors harbor a cell wall sorting signal (CWSS) needed for sortase-mediated pilus assembly, typically comprise a pilus shaft and a tip adhesin. How a pilin becomes a pilus tip, nevertheless, remains undetermined. We demonstrate here in *Actinomyces oris* that the CWSS of the tip pilin CafA is necessary and sufficient to promote pilus tip assembly, and this functional assembly involves a conserved FLIAG motif within the CWSS. This is evidenced by the fact that an *A. oris* cell-wall anchored glycoprotein, GspA, or a heterologous shaft pilin from *Corynebacterium diphtheriae*, SpaA, engineered to have the CWSS of CafA in place of their CWSS, localizes at the pilus tip in a process that requires the FLIAG motif. Our findings provide the molecular basis for sortase-catalyzed pilus tip assembly that is very likely employed by other Gram-positive bacteria and potential bioengineering applications to display antigens at controlled surface distance.

## INTRODUCTION

A variety of multimeric pilus fibers or fimbriae are displayed by many Gram-positive bacteria that include notable pathogens such as *Corynebacterium diphtheriae*, *Actinomyces oris, Bacillus cereus*, *Enterococcus faecalis*, *Streptococcus agalactiae*, *Streptococcus pneumoniae*, and *Streptococcus pyogenes* (1–8). These thread-like polymers are made of shaft pilins covalently linked to a specific tip adhesin, and the resultant tip-containing shafts are linked to a base pilin that is ultimately attached to the cell wall peptidoglycan (9). Pilus polymerization, i.e., the sequential cross-linking of individual pilins to each other, is mediated by a conserved transpeptidase enzyme, named pilus-specific sortase that was first reported in *C. diphtheriae* pili (1). Among the three distinct types of hetero-trimeric pilus structures expressed by *C. diphtheriae* (1, 10, 11), the most studied is the SpaA-type pilus, which comprises the tip adhesin SpaC, the shaft pilin SpaA, followed by the pilin SpaB (1), which serves as base to anchor the pilus shaft to peptidoglycan (12). Each of SpaABC pilins harbors an N-terminal signal peptide for their transport across the cell membrane mediated by the secretion (Sec) machine and a C-terminal cell wall sorting signal (CWSS) for recognition and the catalysis of transpeptidation by sortase. These membrane-bound sortase enzymes form the pilus assembly center that is colocalized with its cognate substrates and the Sec machine (13). Present in all Gram-positive cell wall-anchored proteins, the CWSS represents a tripartite domain that consists of an LPXTG motif, followed by a stretch of hydrophobic amino acids and a tail with positively charged residues (14). Unlike SpaB and SpaC, SpaA uniquely harbors a pilin motif, in which a conserved lysine residue, K190, acts as the essential nucleophile for pilus crosslinking reactions since alanine-substitution of K190 abrogates pilus polymerization (1). Consistent with this, a heterologous protein, *Staphylococcus aureus* enterotoxin B (SEB), engineered to harbor the pilin motif and CWSS of SpaA, is actively polymerized by the pilus-specific sortase SrtA when expressed in *C. diphtheriae*, and this SEB polymerization is abolished with K190A mutation (15). In addition, overexpression of the shaft pilin SpaA results in increased pilus crosslinking reactions, hence pilus length (1), suggesting the cellular availability of substrates and sortase enzymes determines the length of the pilus shaft. According to the current model (9), pilus assembly begins with the cytoplasmic synthesis and translocation of Spa pilin precursors across the cytoplasmic membrane, where oxidatively folded and membrane embedded Spa pilins are captured by the membrane-bound sortase SrtA. SrtA catalyzes pilus polymerization, first linking SpaC to SpaA and then individual SpaA pilins via sequential transpeptidation between the threonine residue of the cleaved LPXTG motif and the conserved lysine residue K190 of the pilin motif. The catalytic cysteine residue of SrtA forms acyl-enzyme intermediates with its cognate substrates, tethering pilus intermediates to the membrane. Pilus polymerization is terminated when SpaB enters the base via a mechanism likely involving substrate competition as SpaB shares structural similarity with the N-terminal domain of SpaA. The pilus polymers with SpaB are then linked to the cell wall by the housekeeping sortase, which also anchors many other surface proteins to peptidoglycan. Although these various steps of pilus assembly have been largely demonstrated through genetic, biochemical, and structural studies (12, 13, 15–21), to date, the molecular determinants and the underlying mechanism that governs orderly assembly of pilus tips remain unknown in *C. diphtheriae* or other Gram-positive bacteria.

Besides *C. diphtheriae*, another important paradigm that helped to reveal the biology of Gram-positive pili and illuminate some of the basic mechanisms that govern pilus assembly is *A. oris*, a prominent member of the human oral biofilms. Unlike the heterotrimeric pili of the *C. diphtheriae* system, *A. oris* produces hetero-dimeric pili that have been classically designated as type 1 and type 2 fimbriae. These fibers, each made of a designated tip pilin and a specific shaft pilin, are encoded by two separate pilus gene clusters harboring pilins and their cognate pilus-specific sortases that are membrane-tethered (2). Although type 1 fimbriae are needed for *Actinomyces* binding to the tooth surface (22), the type 2 fimbriae are required for biofilm formation and polymicrobial interaction, termed coaggregation (23, 24). The type 1 fimbriae, built by the pilus-specific sortase SrtC1, contain FimQ tip and FimP shaft (25), whereas type 2 fimbriae, constructed by SrtC2, contain FimB tip and FimA shaft (2). Remarkably, some of type 2 fimbriae do not contain FimB; these distinct fimbriae are made of a different tip pilin, CafA, attached to the FimA shaft (26). Notably, although deletion of either *fimB* or *cafA* does not severely diminish pilus assembly, the deletion of both *fimB* and *cafA* genes abolishes FimA assembly (26). CafA is one of 14 predicted cell wall-anchored proteins (Aca) in *A. oris* (26), whose genes are not in close proximity with pilin and cognate sortase genes typically found in all Gram-positive pilus gene clusters reported to date (27, 28). Intriguingly, the CWSS of CafA is highly similar to the CWSS of FimB, particularly harboring the FLIAG motif that is absent from the other 13 Aca proteins (26), including AcaC which has been renamed as GspA (29, 30). A highly expressed glycoprotein, GspA is glycosylated by the glycosyltransferase LcpA (29, 30) and anchored to the cell wall by the housekeeping sortase (29). The high degree of sequence similarity between the CWSS of CafA and FimB and the conservation of the FLIAG motif within it lead to the hypothesis of molecular mimicry pinpointing the CWSS as a distinguishing and contributing factor for pilus tip assembly of Gram-positive surface proteins (26, 31)—a plausible conjecture that has remained untested to date.

Here, we demonstrate that the CWSS of CafA is not only necessary for its assembly at the pilus tip in *A. oris*, but it is also sufficient for directing and localizing to the pilus tip of a non-pilus surface protein or even a heterologous pilus shaft protein. Specifically, we first demonstrate that the CWSS of FimB and CafA is interchangeable. We then show that the recombinant non-pilus glycoprotein GspA with its CWSS replaced by the CWSS of CafA is localized at the tip of the FimA pilus shaft, and this pilus tip assembly requires the FLIAG motif. Remarkably, we demonstrated that a heterologous hybrid protein, the SpaA shaft pilin of *C. diphtheriae* with its CWSS swapped by the CafA CWSS, when expressed in *A. oris*, is localized at the tip in a FLIAG-dependent manner. The work presented here provides the molecular basis for sortase-catalyzed pilus tip assembly that is very likely employed by other Gram-positive bacteria as their tip pilins harbor a CWSS. Our findings reported here also have important implications for potential bioengineering applications with the goal to display antigens at controlled surface distance.

## RESULTS

### The cell wall sorting signals of *A. oris* tip pilins FimB and CafA are interchangeable

**Fig 1:**
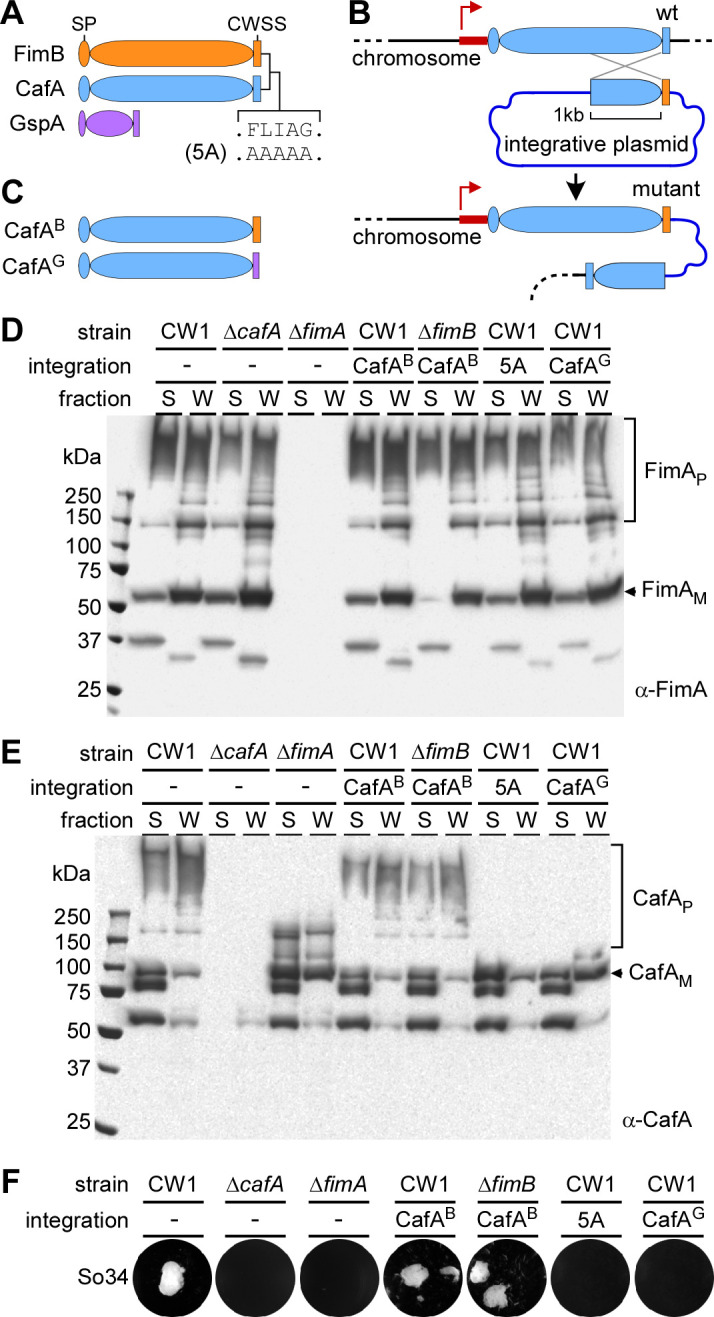
The cell wall sorting signals of FimB and CafA are interchangeable. (**A**) Shown are color-coded diagrams of FimB, CafA, and GspA, with their signal peptide (SP) and cell wall sorting signal (CWSS) drawn in oval and rectangle, respectively. Alanine-substitution of the FLIAG motif shared between FimB and CafA is indicated as 5A. (**B, C**) Depicted is a single-copy gene editing method of replacing the CWSS of endogenous CafA with that of FimB via homologous recombination. An integrative plasmid bearing a homologous sequence joined to a mutated sequence is introduced into *A. oris*, yielding a co-integrant strain that carries a non-functional fragment of CafA. (**D, E**) Cultures of indicated strains grown to mid-log phase were normalized and subjected to cell fractionation. Protein samples obtained from culture supernatant (S) and cell wall (W) fractions were analyzed by SDS-PAGE and immunoblotted with antisera raised against FimA, α-FimA (**D**), and CafA, α-CafA (**E**). The monomers (M) and polymers (P) of FimA and CafA, as well as molecular weight markers (kDa), are indicated. (**F**) Polymicrobial interaction, or coaggregation, was performed with *A. oris* cells mixed with an equivalent number of *S. oralis* So34 cells prior to imaging.

Since the alternate tip pilins CafA and FimB of type 2 fimbriae share a striking similarity in their CWSS, particularly the FLIAG motif adjoining the LPXTG motif (26) (Fig. S1A), we first sought to determine whether their CWSS is interchangeable or not by engineering hybrid proteins (Fig. 1A). Of note, a similar motif was found within the CWSS of some other Gram-positive pilus tip proteins (Fig. S1B). Because CafA overexpression from a multicopy-plasmid appears to reduce pilus assembly (26), we employed a single-copy gene editing approach recently developed for *A. oris* to generate chromosomal mutations or sequence substitutions in CafA (32). In this method, illustrated in Fig. 1B, a DNA fragment with sequence alteration in the desired part of CafA was cloned in an integrative plasmid, along with a 1 kb flanking homologous sequence for recombination; the recombinant plasmid was next used to generate by simple one-step chromosomal integration the intended hybrid *cafA* gene, followed by a promoter-less *cafA* fragment. Using this method, we generated strains expressing CafA hybrid with the CWSS of FimB (CafA^B^) from the natural promoter in the wild-type background (CW1) or an *A. oris* mutant devoid of *fimB* (∆*fimB*) (Fig. 1C). In addition, a strain expressing a CafA hybrid protein (CafA^G^) harboring the CWSS of GspA, a non-pilus cell wall anchored glycoprotein (29), was constructed to serve as a control (Fig. 1C). Cultures of this collection of *A. oris* strains, harboring wild-type or hybrid CafA genes, were then subjected to cell fractionation, and protein samples from the cultural supernatant (S) and cell wall (W) fractions were analyzed by immunoblotting with polyclonal antibodies against FimA (α-FimA), CafA (α-CafA), or GspA (α-GspA). The immunoblot with α-FimA showed that compared with the parent strain CW1—where abundant amounts of high molecular mass FimA polymers were present in the cell wall fraction, with some polymers also detected in the extracellular milieu—no significant changes in this pattern of polymerization and cell wall anchoring of FimA polymers were observed in strains expressing the CafA hybrid proteins (Fig. 1D). Remarkably, the immunoblot with α-CafA demonstrated that strains expressing CafA with the CWSS of FimB were able to incorporate hybrid CafA into FimA polymers (Fig. 1E; compare lanes CafA^B^ with lanes—in CW1), while the strain expressing CafA with the CWSS of GspA displayed only monomers anchored to the cell wall (Fig. 1E, last two lanes, CafA^G^). Importantly, alanine-substitution of the FLIAG motif (the mutant designated as 5A) significantly abrogated CafA incorporation into pilus polymers, while the monomeric form of CafA (CafA_M_) was still functional and capable of anchoring to the cell wall (Fig. 1E; two penultimate lanes). As expected, neither glycosylation nor cell wall anchoring of the native glycoprotein GspA was altered in these strains (Fig. S1C).

Because optimal localization of CafA at the pilus tip is required for polymicrobial interaction, or coaggregation (26, 33), we next examined whether the CafA hybrid proteins are properly displayed and functional using a standard coaggregation assay (33). In this procedure, *A. oris* cells were mixed in equal volumes with *Streptococcus oralis* (So34) cells of similar density, and coaggregation was imaged after a few minutes of gentle shaking. As shown in Fig. 1F, the *A. oris* strains expressing CafA with the CWSS of FimB (CafA^B^) adhered to So34 at the level similar to that of the parent CW1 strain. In sharp contrast, the CafA^5A^ mutant and CafA^G^ strains failed to interact with streptococci, in spite of CafA being anchored to the cell wall. The results indicate that the CWSSs of two tip pilins FimB and CafA are interchangeable and that the FLIAG motif is critical for CafA incorporation into pili but dispensable for cell wall anchoring.

### The cell wall sorting signal of *A. oris* tip pilins is required for pilus tip incorporation

**Fig 2:**
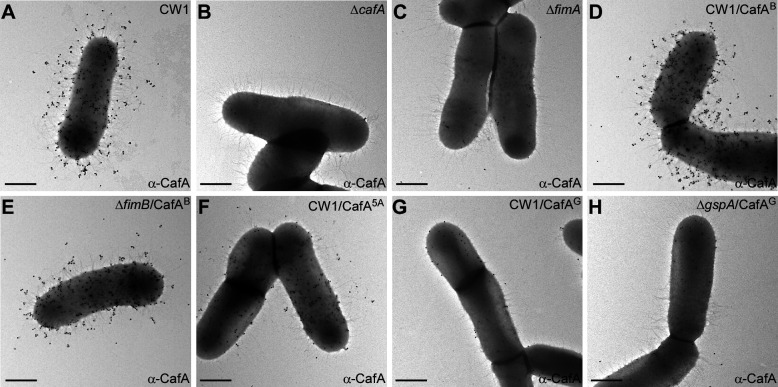
Pilus tip assembly of hybrid CafA is analyzed by immunoelectron microscopy. (**A–H**) Cells of indicated *A. oris* strains were immobilized on carbon-coated nickel grids, treated with α-CafA and labeled with 18 nm gold particles conjugated to IgG; scale bar of 0.5 µm.

To determine whether the CafA hybrid protein polymers presented above is the result of proper pilus tip incorporation of CafA, we performed immunoelectron microscopy (IEM) of the aforementioned strains according to a published protocol (33). Accordingly, *A. oris* cells isolated from normal cultures were stained with α-CafA, followed by gold particles conjugated to IgG prior to electron microscopic analysis. Similar to the parent CW1 strain, *A. oris* strains expressing the CafA hybrid protein with the CWSS of FimB (CafA^B^) abundantly assembled pilus tips displaying CafA, and the deletion of *fimB* did not affect CafA hydrid’s pilus tip incorporation (Fig. 2; compare panels D and E with panel A). The proper tip localization of hybrid CafA^B^ was further confirmed by double labeling IEM with individual antibodies α-CafA and α-FimA (Fig. S2; panels A and B). In sharp contrast, the mutant strain expressing CafA with alanine-substitution of the FLIAG motif (CafA-5A) produced drastically fewer pili with tip-localized CafA, while the non-pilus surface localized CafA was abundantly observed (Fig. 2; compare panel F with panel A). Significantly, no tip localization of the CafA hybrid protein with the CWSS of GspA was detected, although the CafA^G^ hybrid protein was still observed on the cell surface at a level similar to that of the mutant Δ*fimA* in which type 2 fimbriae are not produced (Fig. 2; compare panels G-H to panel C). Consistent with the role of FimA as the major shaft pilin of type 2 fimbriae, surface assembly of FimA polymers was largely unaffected by the presence of CafA hybrid proteins, as determined by IEM (Fig. S3). We conclude that the CWSSs of CafA and FimB are necessary for their pilus tip localization, and the FLIAG motif is critical for this process.

### The cell wall sorting signal of CafA is sufficient to promote pilus tip incorporation of a non-pilus cell surface protein

**Fig 3:**
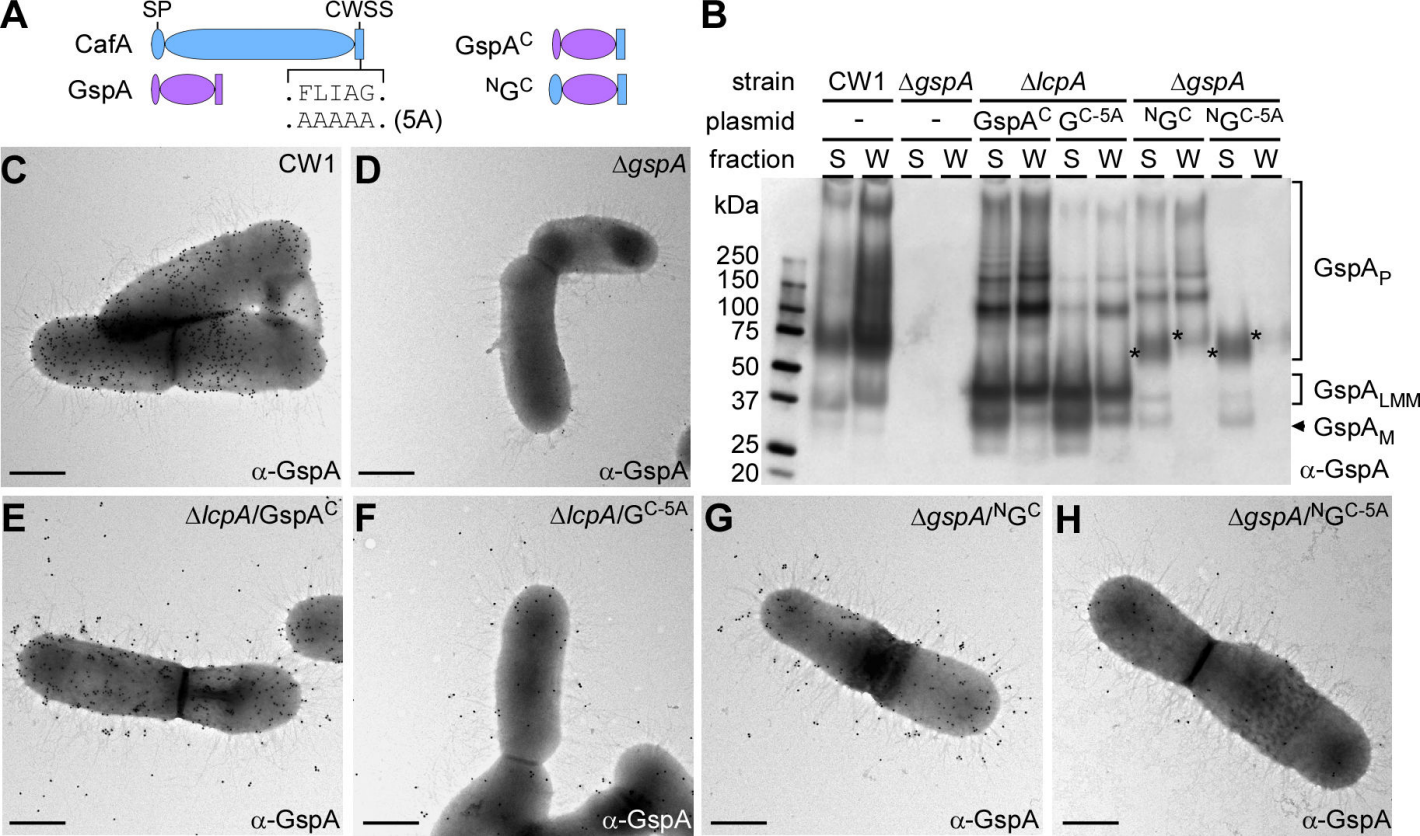
The CWSS of CafA promotes the pilus tip assembly of an *A. oris* non-pilus protein. (**A**) Shown are diagrams of native CafA, GspA, and GspA hybrid proteins harboring the CWSS of CafA (GspA^C^) or the SP and CWSS of CafA (^N^G^C^). (**B**) Protein samples from the culture supernatant (S) and cell wall (W) fractions of indicated strains grown to mid-log phase were immunoblotted with polyclonal antibodies against GspA (α-GspA). The monomers (M), polymers (P), and low molecular mass (LMM) species of GspA, as well as molecular weight markers (kDa), are specified. Asterisks mark the glycosylated forms of GspA in this background strain. (**C-I**) Immunoelectron microscopy of indicated strains was performed, as similarly described in Fig. 2, using α-GspA and 18 nm gold particles conjugated to IgG; scale bar of 0.5 µm.

To determine whether the CWSS of CafA can promote pilus tip incorporation of a normally non-pilus cell surface protein, we selected *A. oris* GspA, which is anchored to the cell wall as a monomeric glycoprotein catalyzed by the housekeeping sortase of *A. oris* (29). We generated four hybrid proteins, in which we replaced the CWSS of GspA with that of CafA (GspA^C^) or both signal peptide (SP) and CWSS of GspA with that of CafA (^N^G^C^), as well as equivalent constructs with the alanine-substitution of the FLIAG motif (Fig. 3A). Of note, the expression of the GspA^C^ constructs is driven by their native *gspA* promoter, whereas the ^N^G^C^ constructs are expressed by the native *cafA* promoter, which is not as highly active as the *gspA* promoter. Using the same single-copy gene editing method described above, we created strains expressing these hybrid proteins in the background of ∆*lcpA* or ∆*gspA*. Various *A. oris* strains were then analyzed by immunoblotting and IEM as described above.

In the parent strain CW1, the immunoblot shows glycosylated GspA (GspA_P_) in the cell wall fraction mainly detected as smear, with a minor amount secreted in the extracellular milieu (Fig. 3B; first two lanes), as reported previously (29). Remarkably, in the ∆*lcpA* mutant, in which GspA is not fully glycosylated, and bands of low molecular mass (GspA_LMM_) and monomeric GspA species (GspA_M_) are typically observed (29, 30), our analysis of the GspA^C^ hybrid revealed distinct GspA polymers, whose migration patterns are visibly different compared with that of the glycosylated GspA polymers (Fig. 3B; ∆*lcpA,* GspA^C^ lanes). By comparison, a significant reduction in the relative amounts of these distinctive bands was observed with the equivalent hybrid construct containing the 5A mutant (Fig. 3B; ∆*lcpA* lanes, compare GspA^C^ lanes and G^C-5A^ lanes). Because GspA is one of the most abundantly produced cell wall anchored proteins in *A. oris* (29, 34), and its membrane translocation might affect its LcpA-independent assembly into pili, we examined whether reduced expression of GspA via the native *cafA* promoter alters GspA pilus incorporation by analyzing the ^N^G^C^ constructs. Strikingly, bands indicative of polymerization of the GspA hybrid protein having both the SP and CWSS of CafA, i.e., ^N^G^C^, was still observed, albeit with a reduced intensity compared with GspA^C^ expressed from its native promoter, and alanine-substitution of the FLIAG motif in ^N^G^C^ abolished these polymers in the cell wall fraction (Fig. 3B; ∆*gspA* lanes and Fig. S4A; ∆*gspA*/∆*lcpA* lanes). Note that the genetic manipulation required to construct these strains did not alter the overall pilus polymerization, cell wall anchoring, and surface assembly of endogenous CafA and FimA (Fig. S4).

Next, to confirm whether the incorporation of hybrid GpsA proteins in polymers as detected in the ∆*lcpA* strain is the result of pilus tip assembly of the GspA hybrid, we analyzed the same sets of strains using IEM. In the parent CW1 strain, GspA signal was observed solely in close apposition to the cell surface, and these signals were absent in the ∆*gspA* mutant (Fig. 3C and D). Strikingly, regardless of the absence or presence of the glycosyltransferase LcpA, GspA hybrid proteins with the CafA CWSS or both promoter-SP and CWSS from CafA were able to locate at the pilus tip (Fig. 3E and G; Fig. S2C and D and S5), and mutations of the FLIAG motif drastically reduced tip localization of the hybrid GspA (Fig. 3F and H; Fig. S5). Altogether, the results demonstrate that the CWSS of CafA is sufficient to promote pilus tip localization of an endogenous cell wall anchored protein and that the FLIAG motif present in the CWSS of CafA plays an essential role in this process.

### The cell wall sorting signal of CafA is sufficient to confer tip identity to a heterologous pilus shaft protein from *C. diphtheriae*

**Fig 4:**
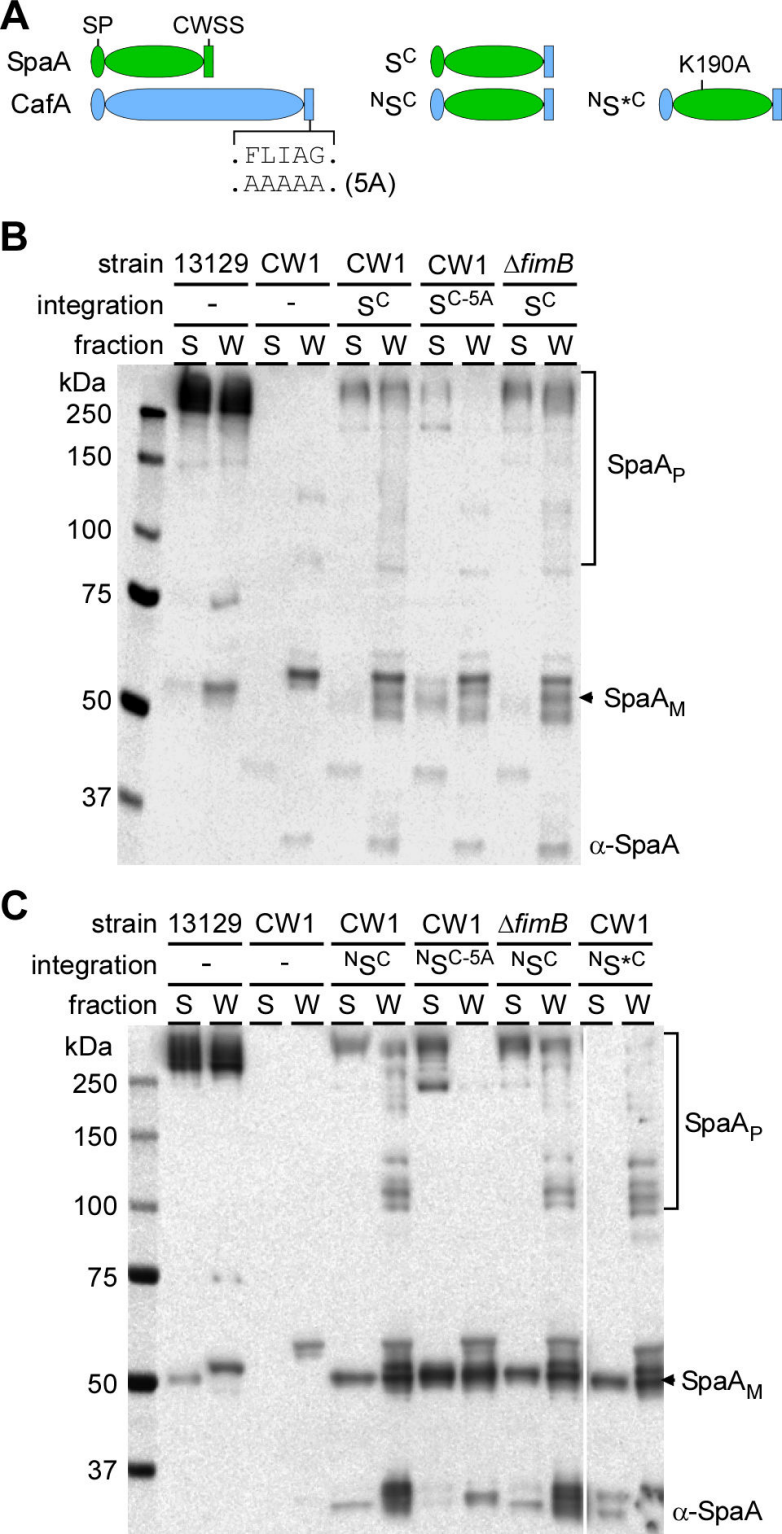
The CWSS of CafA mediates pilus tip assembly of a *C. diphtheriae* pilus shaft protein. (**A**) Graphic presentations of *C. diphtheriae* SpaA and *A. oris* CafA are shown, along with the hybrid SpaA proteins with its CWSS replaced by CafA (S^C^), its SP and CWSS swapped for that of CafA (^N^S^C^), or this protein with K190A mutation (^N^S*^C^). (**B-C**) Normalized cell cultures of *C. diphtheriae* strain 13129 and *A .oris* strains harboring various SpaA constructs were subjected to cell fractionation and immunoblotting with polyclonal antibodies against SpaA (α-SpaA), as similarly described in Fig. 3B, with SpaA monomers and polymers indicated.

As a further test for the sufficiency of CWSS of a tip pilin for its pilus tip destination, we determined whether the CWSS of CafA can confer tip pilin identity to a heterologous protein. We chose the well-studied SpaA pilin from *C. diphtheriae* that normally serves to build a pilus shaft in this organism. Similar to experiments performed above with GspA hybrids, we constructed a hybrid SpaA protein with its CWSS replaced by that from CafA (Fig. 4A; S^C^) and another hybrid protein with both SP and CWSS of SpaA replaced by those of CafA (Fig. 4A; ^N^S^C^). These hybrid proteins were expressed in the background of the *A. oris* strain CW1 and its derivative ∆*fimB*, and their localization was analyzed by western blotting and IEM.

As a major shaft pilin of *C. diphtheriae*, SpaA is polymerized into high molecular mass polymers (SpaA_P_) that are cell wall anchored, with some polymers secreted into the extracellular milieu as detected by immunoblotting with polyclonal antibodies against SpaA, α-SpaA (1, 20) (see also Fig. 4A; lanes 13129, our reference *C. diphtheriae* strain). Strikingly, compared with the *A. oris* parent strain CW1 in which the SpaA_P_ signal was absent, the introduction of SpaA hybrid protein S^C^ resulted in the production of high molecular mass polymers largely recovered in the cell wall fraction, albeit a minor fraction secreted into the culture medium (Fig. 4B; compare lanes CW1/S^C^ to lanes CW1). Alanine-substitution of the FLIAG motif in this SpaA hybrid abrogated the cell wall-anchored SpaA_P_ species but surprisingly not the secreted one (Fig. 4B; lanes CW1/S^C-5A^). As expected, deletion of *fimB* did not affect formation of SpaA_P_ and the cell wall anchoring of the polymers (Fig. 4B; last two lanes).

The results observed for SpaA_P_ polymer species with the ^N^S^C^ constructs expressed in the same set of strain backgrounds just mentioned closely mirrored those of SpaA_P_ with the S^C^ constructs (Fig. 4B and C). Moreover, both S^C^ and ^N^S^C^ constructs with mutations of the FLIAG motif produced some high molecular mass SpaA_P_ that were secreted in the culture medium (Fig. 4B and C; S lanes, CW1/S^C-5A^, and CW1/^N^S^C-5A^). Because SpaA is normally a shaft pilin that harbors the cross-linking pilin motif, we suspected that the SpaA hybrid proteins with an intact pilin motif might also be polymerized into pilus shafts but failed to be anchored to the cell wall. Indeed, with a SpaA point mutant that is unable to crosslink, K190A (15) (^N^S*^C^), the secretion of high molecular mass SpaA_P_ in the culture medium was abrogated, while high molecular mass SpaA_P_ was still detected in the cell wall fraction (Fig. 4C; last two lanes). Note further that formation of FimA polymers was not affected by the presence of the SpaA hybrid proteins in all tested strains (Fig. S6).

**Fig 5:**
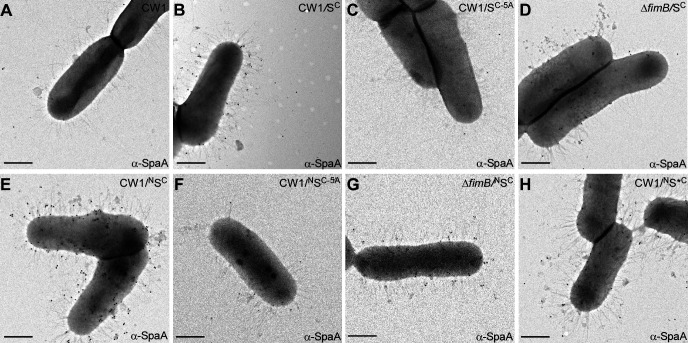
Pilus tip assembly of *C. diphtheriae* SpaA with the CafA CWSS is analyzed by immunoelectron microscopy. (**A-J**) Similar to Fig. 3, indicated *A. oris* cells were subjected to IEM with α-SpaA and 18 nm gold particles conjugated to IgG; scale bar of 0.5 µm.

Finally, to demonstrate that the SpaA hybrid protein is in fact localized at the pilus tip and it serves to nucleate the assembly of the FimA shaft, we employed IEM using α-SpaA as described above. In contrast to the parent strain CW1 that lacks the *spaA* gene and hence displayed no signal of SpaA, the same strain expressing the SpaA hybrid protein displayed abundant SpaA signals at a distance from the cell surface, and the same result was seen with the ∆*fimB* mutant expressing S^C^ (Fig. 5; compare panels B and D to panel A; Fig. S2E). Alanine-substitution of the FLIAG motif virtually abolished detectable cell surface signals for SpaA (Fig. 5C). Furthermore, similar phenotypes of tip localization with SpaA harboring both SP and CWSS of CafA were observed in the same set of strains (Fig. 5E through G; Fig. S2E). Consistent with the result in Fig. 4C, SpaA tip localization was still observed when the pilin motif was inactivated by K190A mutation (Fig. 5H; Fig. S2F). We conclude that the CWSS of a tip pilin is necessary and sufficient to convert the identity of a heterologous shaft pilin to that of the tip pilin maintaining the native specificity for the cognate pilus-specific sortase.

## DISCUSSION

The display of cell surface adhesins at a pilus tip is a common feature of bacterial pili. Well-known examples of pilus tip adhesins include PapG and FimH of *Escherichia coli* chaperone/usher (CU) pili (35, 36), FimC and FimD of *Porphyromonas gingivalis* type V pili (37), and *C. diphtheriae* SpaC, *A. oris* CafA, and *S. pyogenes* AP1 of sortase-assembled pili (26, 38, 39). The mechanism by which a tip pilin is selected in the chaperone/usher pathway has been revealed for *E. coli*. In this system, formation of the tip fibrillum of the P pilus is based on the differential affinity of pilin subunits for the usher PapC, which permits the preferential capture of the chaperone-adhesin PapD-PapG complex to empty PapC sites, followed by PapD-PapF, PapD-PapE, and PapD-PapK complexes (40). Yet another mechanism in Gram-negative bacteria has been suggested for *P. gingivalis*, in which the assembly of the tip adhesin and the pilus stalk occurs through a strand-exchange process by which the order of subunit assembly may rely on a chaperone and/or other factors (41). To date, however, how Gram-positive adhesins such as SpaC and CafA are assembled at the pilus tip has not been revealed. Here, we demonstrate that the CWSS of a tip pilin in *A. oris* is key to the process of pilus tip assembly.

Our previous study of a paradoxical observation that a mutant devoid of the major shaft pilin FimA was completely defective in polymicrobial interaction, or coaggregation (24). However, polyclonal antibodies against FimA or its cognate tip pilin FimB failed to block this coaggregation process (26), leading to the discovery of the major coaggregation factor CafA in *A. oris* (26). That CafA is not genetically linked to the *fimAB* pilus gene cluster but is localized at the tip of FimA fibers in lieu of and independent of FimB (26) has provided a convenient system to address the questions as to how sortases select a tip pilin during pilus assembly. Because CafA and FimB have a highly similar CWSS containing a FLIAG motif, we hypothesized that a molecular mimicry involving the CWSS might enable CafA to substitute FimB as the tip pilin for assembly of the FimA shaft. Indeed, we show here that CafA with its CWSS replaced by that of FimB is still localized at the pilus tip and able to mediate coaggregation as the wild-type, whereas another CafA hybrid protein with the CWSS of glycoprotein GspA is unable to do so (Fig. 1 and 2; Fig. S2). We further demonstrate that the FLIAG motif of the CWSS plays a critical role in the tip assembly of CafA, but not its anchoring to the cell wall, since CafA is still found anchored to the cell wall when its FLIAG motif is substituted with alanine residues (Fig. 1 and 2; Fig. S2). Note that the cell wall anchored CafA mutant protein is unable to mediate coaggregation (Fig. 1F) since this process requires CafA located at an optimal distance away from the cell surface that is otherwise provided by pilus tip localized CafA (33).

Critical pieces of evidence that the CWSS itself governs pilus tip assembly came from our engineering and *in vivo* analysis of two sets of hybrid proteins derived from GspA of *A. oris* and SpaA of *C. diptheriae*. GspA is an abundant surface protein of *A. oris* that is glycosylated by the glycosyltransferase LcpA prior to anchoring to the bacterial cell wall via its CWSS recognized by the housekeeping sortase (29, 30). Remarkably, when the CWSS of GspA is replaced by that of CafA (GspA^C^ and ^N^G^C^ constructs), the hybrid GspA proteins are localized at the pilus tip regardless of the presence or absence of LcpA through a sorting process involving the FLIAG motif (Fig. 3; Fig. S2 and S5). To our knowledge, this provided the evidence for the first time that the CWSS of a tip pilin can convert an otherwise non-pilus surface protein to a pilus tip protein. For this to occur, the tip pilin CWSS in the hybrid protein is not only recognized by the pilus-specific sortase but also enables the nucleation of correct pilus shaft assembly with the bona fide shaft pilin catalyzed by this sortase. In other words, the determinants that govern the identity of a tip pilin in Gram-positive bacteria are encoded within the CWSS of the pilin that is directly recognized by the cognate pilus-specific sortase. It will be important to illuminate the underlying molecular mechanisms by which the various features of a tip pilin CWSS and the FLIAG motif serve to direct the cognate sortase to initiate pilus assembly by joining a shaft pilin monomer to the tip pilin.

To further establish our inference that the CWSS of a tip pilin is necessary and sufficient for initiating pilus assembly by a sortase, we proceeded to extend our experimental approach by using a completely heterologous protein. The chosen protein in this case is a pilin from *C. diphtheriae*, SpaA, which normally constitutes the pilus shaft of one of three distinct types of pili from this organism, whose polymerization requires a pilin motif containing a conserved lysine involved in the formation of the isopeptide bonds that cross-link the shaft pilin to the tip pilin and to itself (1, 15). Indeed, similar to the results obtained with the GspA-CafA hybrid, the SpaA hybrid protein with its own CWSS substituted by that of CafA assembles at the tip of FimA pili in a FLIAG-dependent manner (Fig. 4 and 5; Fig. S2). Interestingly, with the SpaA hybrid that contains the FLIAG motif mutant, we detected some SpaA polymers in the supernatant fractions, which represent secreted pili that failed to anchor to the cell wall (Fig. 4). This observation led us to suspect that some hybrid SpaA monomers might be polymerized into a pilus shaft in addition to serving as the pilus tip. Indeed, alanine-substitution of K190 still maintains the tip assembly of hybrid SpaA protein, while abolishing secreted SpaA polymers (Fig. 4). In this context, it is noteworthy that *A. oris* FimA is highly homologous to SpaH (2), the pilus shaft of SpaH-type pili in *C. diphtheriae* (1, 10), and that FimA can be polymerized by sortase SrtD (15), a pilus-specific sortase for the SpaH pili. It thus seems plausible that the pilus-specific sortase SrtC1 is responsible for polymerization of the SpaA hybrid proteins in *A. oris*, since SpaA is strongly similar to FimP (2), the shaft pilin of type one fimbriae, whose polymerization requires SrtC1 (25). Nevertheless, the fact that the K190A mutant of the SpaA hybrid protein locates to the FimA pilus tip in a FLIAG-motif dependent manner demonstrates unequivocally that the CWSS of a tip pilin suffices to localize a protein to the pilus tip.

In conclusion, we reported here the fundamental role of the cell wall sorting signal in dictating the order with which sortases polymerize pilus fibers in Gram-positive bacteria from distinct pilin monomers serving as the tip and the shaft. Our demonstration that the CWSS of tip pilins suffices for pilus tip assembly of heterologous proteins provides a powerful experimental system for biotechnological applications for ordered assembly of designer polymers and display of antigens and modulatory proteins, especially for those that need to be at a critical distance away from the cell surface for their efficient action. A wealth of new insights on the biological mechanisms and tools for precision bioengineering are likely to emerge from further molecular dissection of several Gram-positive bacterial model systems that have already been characterized and accessible to the community.

## MATERIALS AND METHODS

### Bacterial strains, plasmids, and media

Bacterial strains and plasmids used in this study are listed in Table S1. *A. oris* strains were grown in heart infusion broth (HIB) or heart infusion agar (HIA) plates at 37°C (or 30°C for efficient gene deletion) and in the presence of 5% CO_2_. *C. diphtheriae* NCTC 13129 was cultured in HIB or on HIA plates at 30°C. *S. oralis* 34 strain was grown on HIA supplemented with 1% glucose at 37°C in an anaerobic chamber. *E. coli* strains were grown on Luria-Bertani (LB) broth or agar at 37°C. The resistant strains of *A. oris* or *E. coli* were grown in the presence of 50 µg/mL kanamycin.

### Genetic manipulation and single-copy gene editing in *A. oris*

#### Generation of non-polar, in-frame deletion mutants

The Δ*gspA* mutant (29) was used to generate a double mutant devoid of *gspA* and *fimB* (Δ*gspA*-Δ*fimB*), whereas the Δ*cafA*-Δ*fimB* mutant (26) was used to create a triple mutant lacking *cafA*, *fimB*, and *gspA* (Δ*cafA*-Δ*fimB*-Δ*gspA*) (Table S1), according to our published protocols (29, 42). Briefly, 1 kb flanking regions upstream and downstream of *fimB* or *gspA* were PCR-amplified with appropriate primers (Table S2) and cloned into the deletion vector pCWU2 (24). The generated plasmids were individually introduced into *A. oris* cells of appropriate mutants (Δ*gspA*-Δ*fimB* or Δ*cafA*-Δ*fimB*-Δ*gspA*) by electroporation, permitting plasmid integration into the bacterial chromosome and generating integrant strains selected by kanamycin-containing HIA plates. The integrant strains were grown without antibiotic selection, allowing the second homologous recombination leading to plasmid excision to formation of wild-type or mutant alleles, which were selected on agar plates containing 2-deoxy-D-galactose and verified by PCR and DNA sequencing.

#### Generation of integrative plasmids

A derivative of the *A. oris* suicide vector pHTT177 (42) in which an HpaI restriction site was inserted for cloning purposes, the integrative plasmid pHTTh was used to construct various integrative plasmids expressing hybrid variants in *A. oris* (Table S1). Each hybrid protein was cloned according to our published protocol (32). Briefly, a 1 kb homologous sequence of a target gene (*cafA*, *gspA*, or *spaA*), used for homologous recombination, was PCR-amplified with appropriate primers (Table S2). The amplicon was then joined to other amplicon(s), with desired sequences for the CWSS or its mutations, by overlapping PCR. The linked DNA fragments were cloned into pHTTh.

#### Generation of chromosomal hybrid variants

To generate chromosomal mutations or sequence replacements of CafA we employed single-copy gene editing recently developed for *A. oris* to generate chromosomal mutations or sequence replacements of CafA (32). Individual integrative plasmids generated in (ii) were electroporated into *A. oris* cells, and co-integrant strains were selected by kanamycin. Strains with co-integration in proximity of the cafA gene (^N^G^C^, S^C^, ^N^S^C^, and their mutant variants) expressed hybrid proteins under the control of the *cafA* promoter, while the *gspA* promoter controls the expression of G^C^ and its variants.

### Cell fractionation and immunoblotting

Cell fractionation was conducted as previously described with some modification (43). Briefly, 4 mL cultures of *A. oris* strains in HIB were maintained at 37°C with shaking to mid-log phase. *A. oris* cells were harvested by centrifugation, and the cell number of all cell suspensions was normalized to an OD_600_ of 1.0 prior to treatment with mutanolysin for 4 h at 37°C. Protein samples were obtained by precipitation with 7.5% trichloroacetic acid and suspended in hot sodium dodecyl sulfate (SDS) and 3 M urea containing-sample buffer. All samples were analyzed by SDS-PAGE electrophoresis using 4%–15% gradient gels and immunoblotting with appropriate antisera; α-CafA (1:4,000), α-FimA (1:20,000), α-GspA (1:5,000) and SpaA (1:10,000).

### Bacterial coaggregation

Coaggregation between *A. oris* and *S. oralis* 34 cells was determined by previously published coaggregation assays (44). Briefly, *A. oris* and *S. oralis* 34 cells were grown in HIB and HIB supplemented with 1% glucose, respectively. Bacterial cells were normalized to an OD_600_ of 2.0, washed, resuspended in coaggregation buffer (150 mM NaCl, 0.1 mM CaCl_2_, 20 mM Tris-HCl, pH 7.4) in a 1:1 ratio using 24-well plates, and agitated by gentle rotational shaking for a few minutes. Coaggregation was recorded by an Imager (ProteinSimple).

### Immunogold-labeling electron microscopy

Preparation of bacterial samples for electron microscopy was followed according to a published protocol with some modification (26). Briefly, *A. oris* strains grown on HIA plates overnight were used to prepare cell suspension in phosphate-buffered saline (PBS). A drop of bacterial suspension was placed onto carbon-coated nickel grids. Washed cells on grids were blocked with 0.1% gelatin in PBS and then incubated with specific antibodies against *A. oris* proteins (α-FimA, 1:100; α-CafA, 1:50; and α-GspA, 1:100; and α-SpaA, 1:100), followed by staining with a secondary antibody conjugated with gold particles (12 nm for FimA and 18 nm for CafA, GspA, and SpaA). The samples were stained with 1% uranyl acetate prior to imaging with a FEI Tecnai 12 transmission electron microscope.

For double-labeling as previously described (45), bacterial cells on grids were first stained with α-CafA, α-GspA or α-SpaA (1:50 dilution), followed by washing and blocking prior to labeling with goat anti-rabbit secondary antibodies conjugated to 18 nm gold particles (Jackson ImmunoResearch) (1:20 dilution). After an extensive wash with water, the samples were stained with α-FimA (1:100), followed by 12 nm gold particles conjugated to IgG (Jackson ImmunoResearch) (1:20 dilution). The samples were stained with 1% uranyl acetate prior to microscopic analysis.

## Data Availability

All data generated in this study are provided in this article, including the supplemental material.

## SUPPLEMENTAL MATERIAL

Supplemental Material mbio.01484-24-s0001.pdf Supplemental figures and tables.

ASM does not own the copyrights to Supplemental Material that may be linked to, or accessed through, an article. The authors have granted ASM a non-exclusive, world-wide license to publish the Supplemental Material files. Please contact the corresponding author directly for reuse.
