# Supplementary material for: Molecular basis for sortase-catalyzed pilus tip assembly
Source: mBio. 2024 Aug 2;15(9):e01484-24. doi: 10.1128/mbio.01484-24 (PMC11389406; doi:10.1128/mbio.01484-24)
Supplement: Supplemental Material — Supplemental figures and tables. [file mbio.01484-24-s0001.pdf]

Molecular basis for sortase-catalyzed pilus tip assembly

Aadil H. Bhat[1], Chungyu Chang[1], Asis Das[2], and Hung Ton-That[1,3,4†]

[1]*Division of Oral & Systemic Health Sciences, School of Dentistry, University of California, Los Angeles, California, USA;* [2]*Department of Medicine, Neag Comprehensive Cancer Center, School of Medicine, University of Connecticut Health Center, Farmington, CT, USA;* [3]*Department of Microbiology, Immunology & Molecular Genetics, University of California, Los Angeles, Los Angeles, CA, USA;* [4]*Molecular Biology Institute, University of California, Los Angeles, California, USA*

[†]To whom correspondence may be address: Hung Ton-That, htonthat@dentistry.ucla.edu

Running Title: *Molecular determinants of pilus tip localization*

Keywords: *Actinomyces oris*, sortase, pilus assembly, tip pilin, secretion, cell wall anchoring

Supporting Figures

Figure S1: Formation of CafA hybrid proteins does not affect glycosylation and cell wall anchoring of GspA. (A) Presented are schematic diagrams of FimB, CafA, and GspA proteins, as well as hybrid constructs, with their signal peptide (SP) and cell wall sorting signal (CWSS) depicted in oval and rectangle, respectively. FimB and CafA share a similar CWSS, with conserved residues highlighted in black boxes. CafA with the FLIAG motif mutated to five alanine residues is denoted as 5A. **(B)** Shown is the alignment of the CWSS sequences obtained from the tip proteins of *Streptococcus pneumoniae* (RrgA), *Streptococcus pyogenes* (FctX), *A. oris* (CafA and FimB), and *Corynebacterium diphtheriae* (SpaG). The alignment was performed using Clustal Omega (1). **(C)** Cultures of indicated strains grown to mid-log phase were normalized and subjected to cell fractionation. Protein samples obtained from culture supernatant (S) and cell wall (W) fractions were analyzed by SDS-PAGE and immunoblotted with antisera raised against GspA, α-GspA. The polymers (P) of GspA and molecular weight markers (kDa) are indicated.

Figure S2: The CWSS of CafA promotes pilus tip assembly of hybrid proteins. (A-F) Cells of indicated *A. oris* strains were immobilized on carbon-coated nickel grids and subjected to double-labeling, first treated with α-CafA (A-B), α-GspA (C-D), or α-SpaA (E-F) and labeled with 18-nm gold particles conjugated to IgG. After extensive wash, the samples were stained with α-FimA (1:100), followed by 12-nm gold particles conjugated to IgG. The samples were then stained with 1% uranyl acetate prior to microscopic analysis; scale bars of 0.2 μm. Enlargement of boxed areas is shown to the right of each panel, with black arrows marking 12-nm gold particles reactive with α-FimA and colored arrows marking 18-nm gold particles reactive with specific primary antibodies (α-CafA, α-GspA, or α-SpaA) highlighted with the same color.

Figure S3: Formation of CafA hybrid proteins does not affect pilus assembly of FimA. (A-H) Cells of indicated *A. oris* strains were immobilized on carbon-coated nickel grids and treated

with α-FimA, followed by 12-nm gold particles conjugated to IgG. The samples were then stained with 1% uranyl acetate prior to microscopic analysis; scale bars of 0.5 μm.

Figure S4. Formation of GspA hybrid proteins does not affect CafA and FimA pilus assembly. (A-B) Similar to the experiments described in Fig. S1C, immunoblotting of protein samples harvested from the indicated strains was performed with α-CafA (B) or α-FimA (B). **(C)** Similar to the experiments described in Fig. S3, cells of indicated strains were subjected to immunolabeling with α-CafA or α-FimA, followed by 18-nm (top panels) or 12-nm (bottom panels) gold particles conjugated to IgG; scale bars of 0.5 μm.

Figure S5: The glycosyltransferase LcpA is dispensable for pilus tip assembly. (A) Similar to the experiments described Fig. S1C, immunoblotting of protein samples harvested from the indicated strains was performed with α-GspA. **(B)** Similar to the experiments described in Fig. S3, cells of indicated strains were subjected to immunolabeling with α-GspA, followed by 18-nm gold particles conjugated to IgG; scale bars of 0.5 μm.

Figure S6: Formation of SpaA hybrid proteins does not affect FimA pilus assembly. (A) Presented are schematic diagrams of *C. diphtheriae SpaA* and *A. oris* CafA, and GspA proteins, as well as hybrid constructs, with their signal peptide (SP) and cell wall sorting signal (CWSS) depicted in oval and rectangle, respectively. Mutants with the FLIAG motif mutated to five alanine residues are denoted as 5A. **(B-C)** Similar to the experiments described Fig. S1C, immunoblotting of protein samples harvested from the indicated strains was performed with α-FimA. The monomers (M) and polymers (P) of FimA and molecular weight markers (kDa) are indicated.

Supporting Tables

Table S1: Strains and Plasmids used in this study

Strains & Plasmids	Description	Reference
Strain		
C. diphtheriae NCTC13129	Wild-type	(2)
S. oralis 34	Cell surface receptor polysaccharide positive	(3)
A. oris MG-1	Wild-type	(4)
A. oris CW1	Isogenic derivative of MG-1; Δ*galK*	(5)
A. oris CW2	Isogenic derivative of CW1; Δ*fimB*	(5)
A. oris AR5	Isogenic derivative of MG-1; Δ*cafA*	(6)
A. oris WU59	Isogenic derivative of CW1; Δ*cafA*-Δ*fimB*	(6)
A. oris WU51	Isogenic derivative of CW1; Δ*gspA*	(7)
A. oris WU72	Isogenic derivative of CW1; Δ*lcpA*	(7)
A. oris AH28	Isogenic derivative of CW1; Δ*gspA*-Δ*fimB*	This study
A. oris AH48	Isogenic derivative of CW1; Δ*gspA*-Δ*lcpA*	This study
A. oris AH1	Co-integrant strain expressing CafA^{B} in CW1	This study
A. oris AH3	Co-integrant strain expressing CafA^{B} in Δ*fimB*	This study
A. oris AH5	Co-integrant strain expressing CafA^{5A} in CW1	This study
A. oris AH14	Co-integrant strain expressing GspA^{C} in CW1	This study
A. oris AH15	Co-integrant strain expressing ^{N}G^{C} in Δ*gspA*	This study
A. oris AH22	Co-integrant strain expressing ^{N}G^{C-5A} in Δ*gspA*	This study
A. oris AH30	Co-integrant strain expressing ^{N}G^{C} in Δ*gspA*-Δ*fimB*	This study
A. oris AH31	Co-integrant strain expressing ^{N}G^{C} in Δ*cafA*-Δ*gspA*-Δ*fimB*	This study
A. oris AH35	Co-integrant strain expressing GspA^{G} in Δ*gspA*	This study
A. oris AH37	Co-integrant strain expressing ^{N}S^{C} in CW1	This study
A. oris AH38	Co-integrant strain expressing ^{N}S^{C} in Δ*cafA*	This study
A. oris AH39	Co-integrant strain expressing ^{N}S^{C} in Δ*fimB*	This study
A. oris AH40	Co-integrant strain expressing S^{C} in Δ*cafA*	This study
A. oris AH41	Co-integrant strain expressing S^{C} in Δ*fimB*	This study
A. oris AH42	Co-integrant strain expressing S^{C} in CW1	This study
A. oris AH43	Co-integrant strain expressing ^{N}S^{C-5A} in CW1	This study
A. oris AH44	Co-integrant strain expressing S^{C-5A} in CW1	This study
A. oris AH47	Co-integrant strain expressing ^{N}S^{*C} (K190A) in CW1	This study
A. oris AH49	Co-integrant strain expressing ^{N}G^{C} in Δ*gspA*-Δ*lcpA*	This study
A. oris AH49	Co-integrant strain expressing ^{N}G^{C-5A} in Δ*gspA*-Δ*lcpA*	This study
Plasmids		
pHTT177	*A. oris* integrative plasmid; Kan^{R}	(8)
pHTTh	Derivative of pHTT177 containing a HpaI site	This study
pCafA^{B}	pHTTh containing ~1kb-homologous *cafA* sequence with the FimB CWSS cloned between HpaI and XbaI sites	This study
pCafA^{5A}	pHTTh containing ~1kb-homologous *cafA* sequence with its FLIAG motif mutated to 5A	This study

pCafA^{G}	pHTTh containing ~1kb-homologous *cafA* sequence with its CWSS replaced by that of GspA	This study
pGspA^{C}	pHTTh containing ~1kb-homologous *gspA* sequence with its CWSS replaced by that of CafA	This study
p^{N}GspA^{C}	pHTTh containing ~1kb-N-terminal homologous *cafA* sequence fused to the SP-less *gspA* sequence with its CWSS replaced by that of CafA	This study
pGspA$^{C\text{-}5A}$	pGspA^{C} with the FLIAG motif mutated to 5A	This study
p^{N}GspA$^{C\text{-}5A}$	p^{N}GspA^{C} with the FLIAG motif mutated to 5A	This study
pSpaA^{C}	pHTTh containing~1kb-N-terminal homologous *cafA* sequence fused to the *spaA* sequence with its CWSS replaced by that of CafA	This study
pSpaA$^{C\text{-}5A}$	pSpaA^{C} with the FLIAG motif mutated to 5A	This study
p^{N}SpaA^{C}	pHTTh containing~1kb-N-terminal homologous *cafA* sequence fused to the SP-less *spaA* sequence with its CWSS replaced by that of CafA	This study
p^{N}SpaA$^{C\text{-}5A}$	^{N}SpaA^{C} with the FLIAG motif mutated to 5A	This study
p^{N}SpaA*^{C}	p^{N}SpaA^{C} with K190A mutation	This study

Table S2: Primers used in the study

Primer	Sequence$^{(a)}$	Application
CafA_intF	GGCGGGCACCGCCGACTTG	pCafA^{B} & pCafA^{G}
CafA_intR	GTACG**TCTAGA**TGTCCCTGGCCAAGCAGGTG	pCafA^{B} & pCafA^{G}
FimBss_oF	*CAAGTCGGCGGTGCCCGCC*TTGCCCCTGACAGGCGGAC	pCafA^{B}
FimBss_R	TCAGTCGAGGTTGCAGTGACG	pCafA^{B}
CafA_sdm1_F	CGCGGCCGCGGCCGCGGTGTCGGCCGCGCTCCCTC	All plasmids with C5A
CafAss_hF	**AAC**TCATGAGTCCAGGGCCCGC	All plasmids with the CafA CWSS
CafA_sdm1_R	GCGGCCGCGGCCGCGGGGGCGGTGCTGGGGATCAC	pCafA^{C} & pCafA^{5A}
GspA_intF2	GTACG**TCTAGA**GCGACTCCCTCGCCTTCAAG	pGspA^{C} & pGspA^{C5A}
GspA_intR	GGGCTTGCCGGAGGTGGAGG	pGspA^{C}
Cafss::gsp_R	*CCTCCACCTCCGGCAAGCCC*ATCCCCTTCACCGGAGGGAG	pGspA^{C}
CafA_spF	CGATCCTCCGGCAGGATTGC	p^{N}G^{C} & p^{N}S^{C}
Caf_dgR	GTACG**TCTAGA**GAAGGTTGCGCTCGAGGCAG	All plasmids with the CafA N-terminus
Gsp::Caf_F	*GCAATCCTGCCGGAGGATCG*AAGATCGCCGATGACCAGCAG	p^{N}GspA^{C}
GspA_ssF	*CAAGTCGGCGGTGCCCGCC*GGCAAGCCCCTGGCCCAGAC	pCafA^{G}
GspA_ssR	**AAC**TCAGGCCTTACGACGACGCAC	pCafA^{G}
SpaA:cafF1	*GCAATCCTGCCGGAGGATCG*ACGAGTATTGCCGTGCATGC	p^{N}S^{C}
SpaA_intR	TTCAAATCCGGCGTTCTTTTTCTTG	pS^{C} & p^{N}S^{C}
Cafss:spA_R	*CAAGAAAAAGAACGCCGGATTTGAA*ATCCCCTTCACCGGAGGGAG	p^{N}SpaA^{C}
CafA_pR	GAAGCGCTACCTCAGTTTTAGG	pSpaA^{C}
SpaA:cafF2	*CCTAAAACTGAGGTAGCGCTTC*ATGACTGCACGGGGGTGTCG	pSpaA^{C}
CafAss_kF	CGAGT**GGTACC**TCATGAGTCCAGGGCCCGC	pSpaA^{C}
Gsp_upF	CAGA**GGTACC**CCGACATGGCCTACTCGCTG	Δ*gspA*-Δ*lcpA*
Gsp_upR	*CTCCGGCGAGTGACTAGGC*GCGTCCAAGACGCATGAGTG	Δ*gspA*-Δ*lcpA*
Lcp_dnF	GCCTAGTCACTCGCCGGAG	ΔΔ*gspA*-Δ*lcpA*
Lcp_dnR	CGAC**TCTAGA**AGCAGATGCTGGAGTCGCTC	Δ*gspA*-Δ*lcpA*

$^{(a)}$ Letters in bold are the restriction sites in the primers; underlined sequences are mutation sequence for site-directed mutagenesis; and italicized sequences represent overlapping portion of the primer.

References

1. Madeira F, Madhusoodanan N, Lee J, Eusebi A, Niewielska A, Tivey ARN, Lopez R, Butcher S. 2024. The EMBL-EBI Job Dispatcher sequence analysis tools framework in 2024. Nucleic Acids Res doi:10.1093/nar/gkae241.
2. Cerdeno-Tarraga AM, Efstratiou A, Dover LG, Holden MT, Pallen M, Bentley SD, Besra GS, Churcher C, James KD, De Zoysa A, Chillingworth T, Cronin A, Dowd L, Feltwell T, Hamlin N, Holroyd S, Jagels K, Moule S, Quail MA, Rabbinowitsch E, Rutherford KM, Thomson NR, Unwin L, Whitehead S, Barrell BG, Parkhill J. 2003. The complete genome sequence and analysis of *Corynebacterium diphtheriae* NCTC13129. Nucleic Acids Res 31:6516-23.
3. Yoshida Y, Ganguly S, Bush CA, Cisar JO. 2006. Molecular basis of L-rhamnose branch formation in streptococcal coaggregation receptor polysaccharides. J Bacteriol 188:4125-30.
4. Mishra A, Das A, Cisar JO, Ton-That H. 2007. Sortase-catalyzed assembly of distinct heteromeric fimbriae in *Actinomyces naeslundii*. J Bacteriol 189:3156-65.
5. Mishra A, Wu C, Yang J, Cisar JO, Das A, Ton-That H. 2010. The *Actinomyces oris* type 2 fimbrial shaft FimA mediates co-aggregation with oral streptococci, adherence to red blood cells and biofilm development. Mol Microbiol 77:841-54.
6. Reardon-Robinson ME, Wu C, Mishra A, Chang C, Bier N, Das A, Ton-That H. 2014. Pilus hijacking by a bacterial coaggregation factor critical for oral biofilm development. Proc Natl Acad Sci U S A 111:3835-40.
7. Wu C, Huang IH, Chang C, Reardon-Robinson ME, Das A, Ton-That H. 2014. Lethality of sortase depletion in *Actinomyces oris* caused by excessive membrane accumulation of a surface glycoprotein. Mol Microbiol 94:1227-41.

8. Wu C, Ton-That H. 2010. Allelic exchange in *Actinomyces oris* with mCherry fluorescence counterselection. Appl Environ Microbiol 76:5987-9.

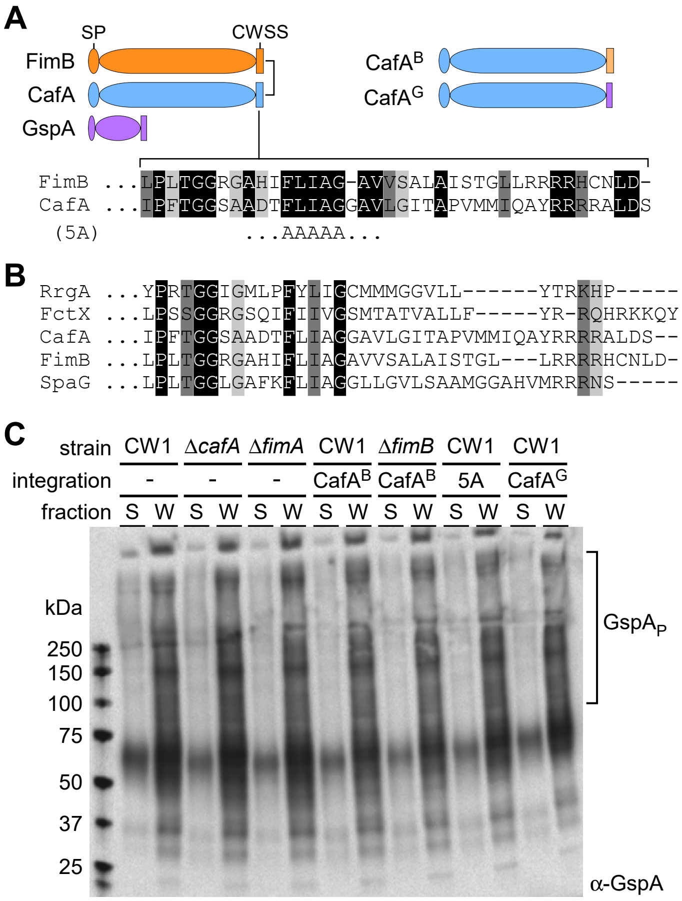

Figure S1: Bhat et al.

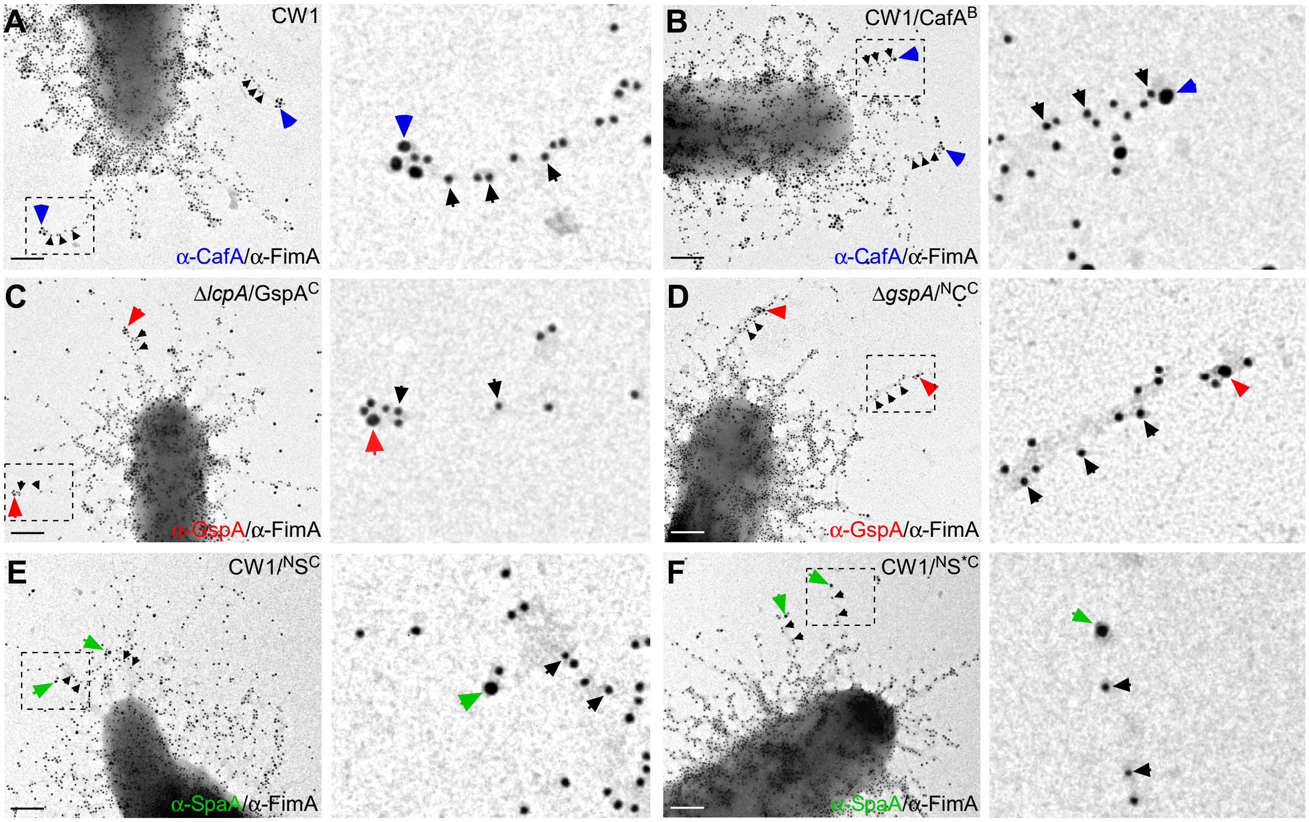

Figure S2: Bhat et al.

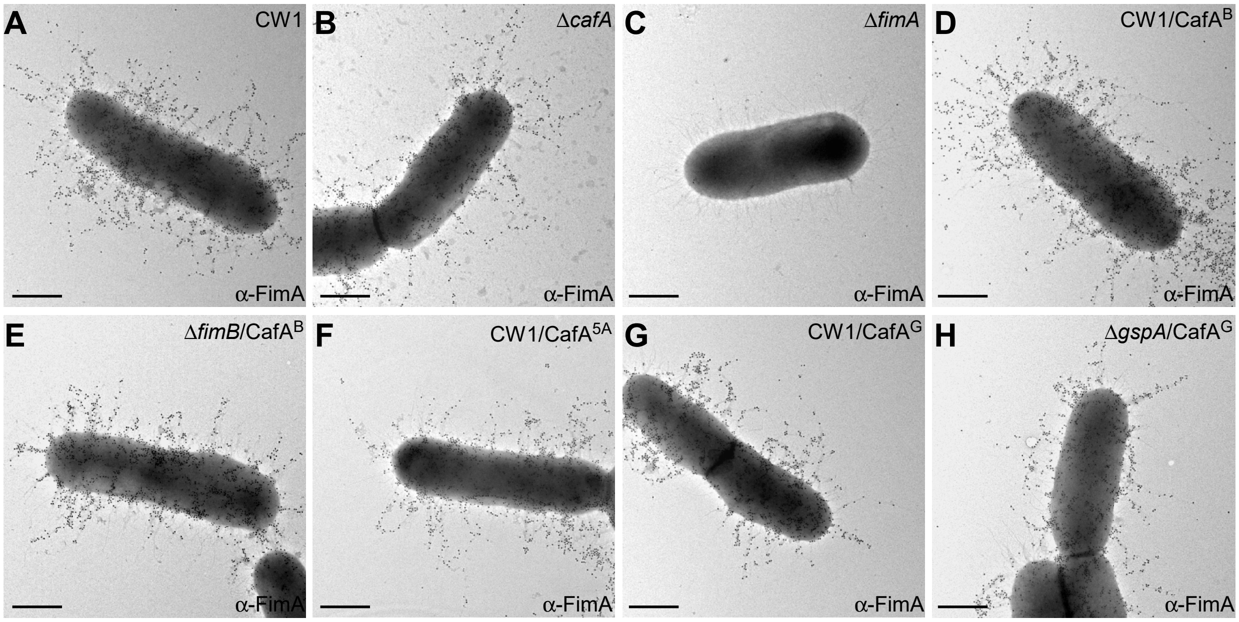

Figure S3: Bhat et al.

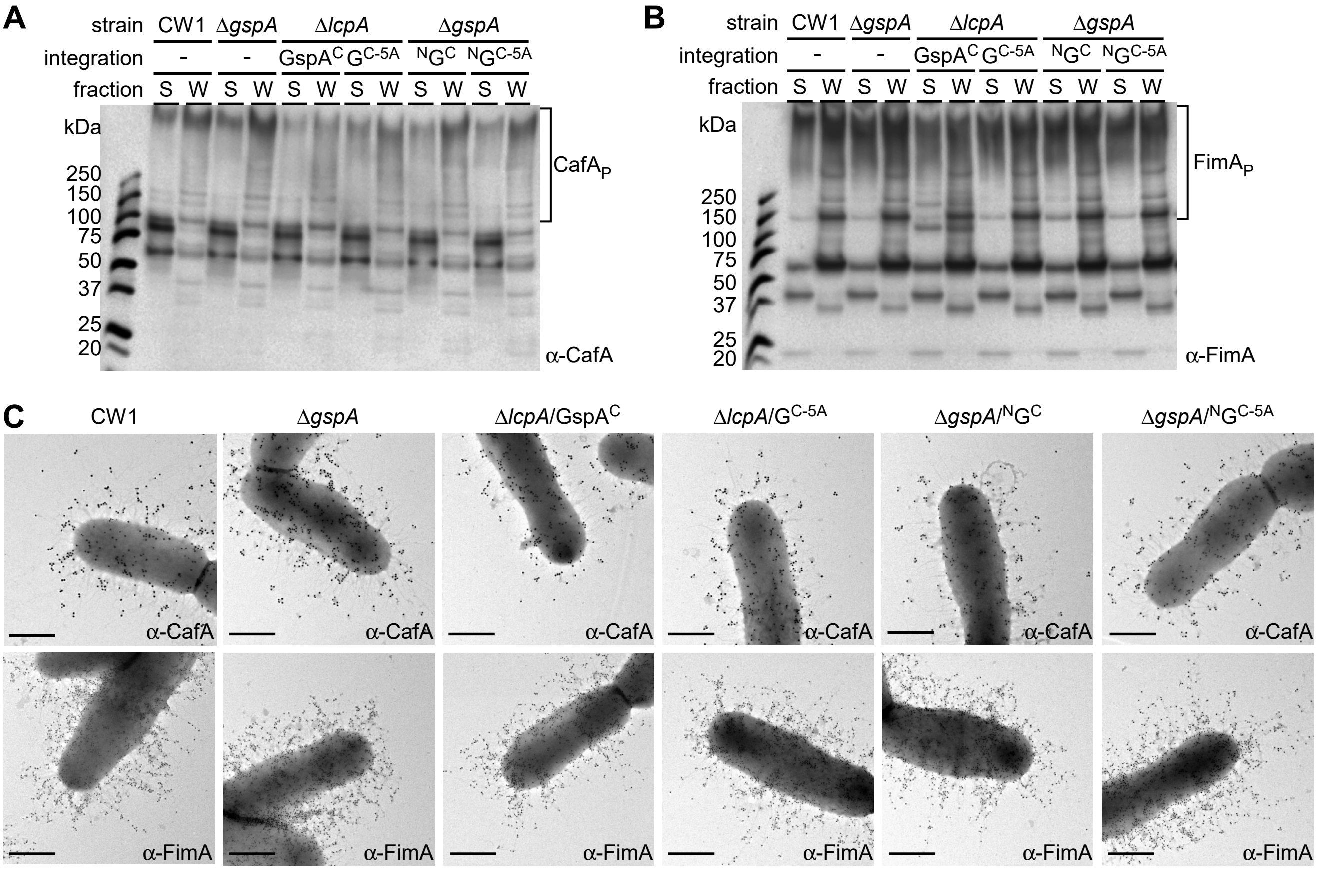

Figure S4: Bhat et al.

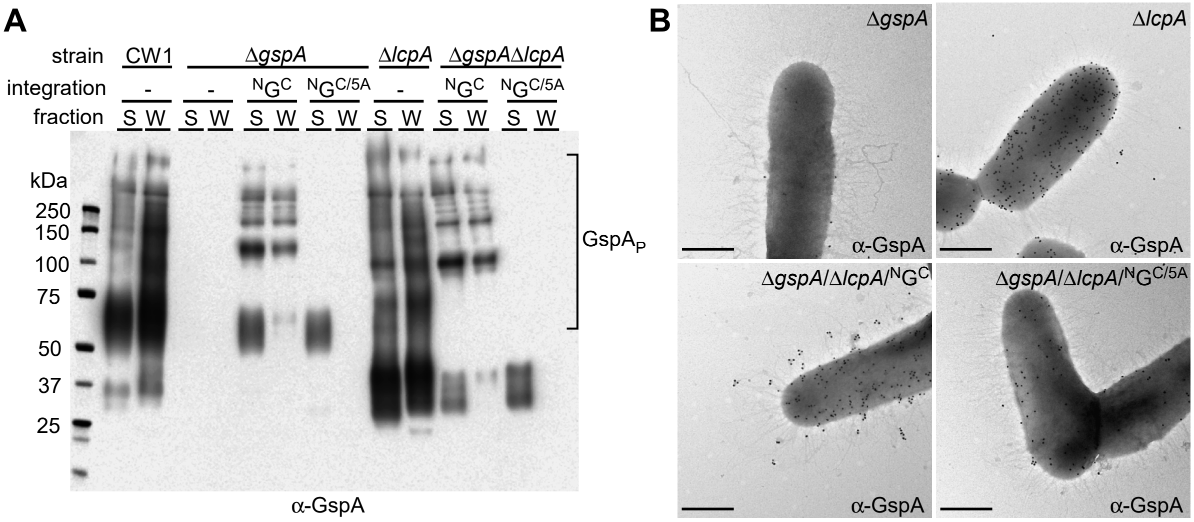

Figure S5: Bhat et al.

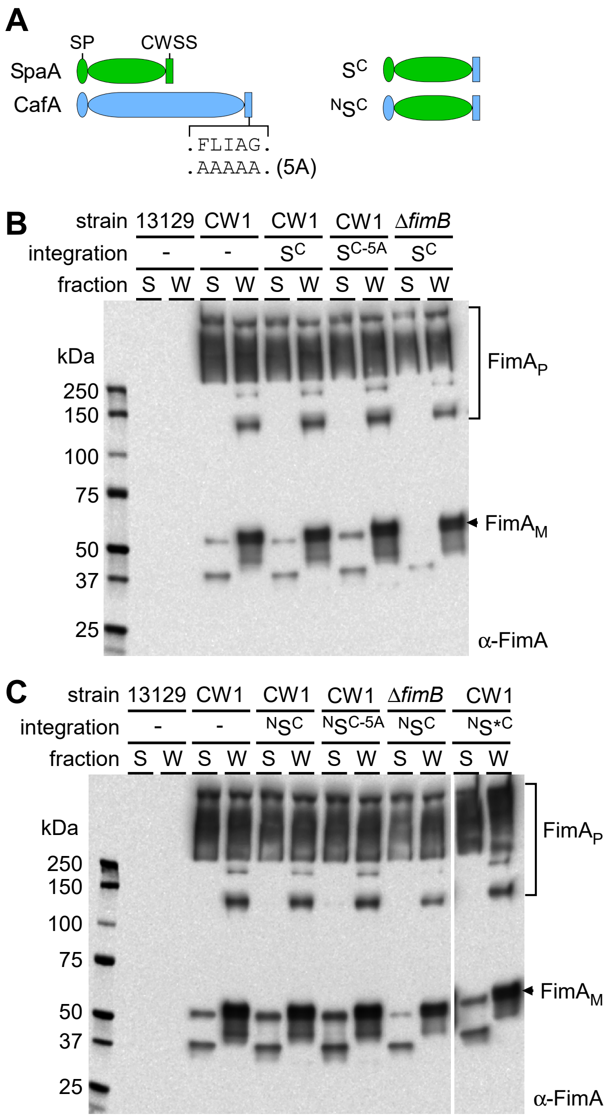

Figure S6: Bhat et al.
